# Apolipoprotein E in Cardiometabolic and Neurological Health and Diseases

**DOI:** 10.3390/ijms23179892

**Published:** 2022-08-31

**Authors:** Jeyashree Alagarsamy, Anja Jaeschke, David Y. Hui

**Affiliations:** 1 Molecular Genetics, Biochemistry and Microbiology Graduate Program, University of Cincinnati College of Medicine, Cincinnati, OH 45237, USA; 2 Department of Pathology, University of Cincinnati College of Medicine, Cincinnati, OH 45237, USA

**Keywords:** apolipoprotein E (apoE), lipoprotein receptors, atherosclerosis, inflammatory response, Alzheimer’s disease, signal transduction

## Abstract

A preponderance of evidence obtained from genetically modified mice and human population studies reveals the association of apolipoprotein E (apoE) deficiency and polymorphisms with pathogenesis of numerous chronic diseases, including atherosclerosis, obesity/diabetes, and Alzheimer’s disease. The human *APOE* gene is polymorphic with three major alleles, ε2, ε3 and ε4, encoding apoE2, apoE3, and apoE4, respectively. The *APOE* gene is expressed in many cell types, including hepatocytes, adipocytes, immune cells of the myeloid lineage, vascular smooth muscle cells, and in the brain. ApoE is present in subclasses of plasma lipoproteins, and it mediates the clearance of atherogenic lipoproteins from plasma circulation via its interaction with LDL receptor family proteins and heparan sulfate proteoglycans. Extracellular apoE also interacts with cell surface receptors and confers signaling events for cell regulation, while apoE expressed endogenously in various cell types regulates cell functions via autocrine and paracrine mechanisms. This review article focuses on lipoprotein transport-dependent and -independent mechanisms by which apoE deficiency or polymorphisms contribute to cardiovascular disease, metabolic disease, and neurological disorders.

## 1. Introduction

The *APOE* gene encoding the apolipoprotein E (apoE) protein is one of the most widely studied genes in biomedical research [1]. Its popularity owes in large part to the suitability of *ApoE^−/−^* mice as an experimental animal model to study the pathogenesis of numerous diseases, as well as the association between *APOE* polymorphism and a wide spectrum of diseases in humans, including coronary and peripheral vascular disease [2,3,4,5,6,7,8,9,10,11,12,13], obesity/diabetes [10,14,15], and Alzheimer’s disease [16]. While no polymorphism in the mouse *ApoE* gene has been reported to date, the human *APOE* gene is polymorphic, with three common alleles designated as ε2, ε3 and ε4, resulting in six genotypes: ε2/2, ε2/3, ε2/4, ε3/3, ε3/4, and ε4/4. The ε3 allele is the most prevalent form, accounting for 70–80% worldwide, while 8% of the population are carriers for the ε2 allele and 14% of the population are ε4 allele carriers. The ε3 allele encodes the apoE3 isoform with a cysteine residue at position 112 and an arginine residue at position 158 of the 299-residue protein. The ε2 allele encodes apoE2 with a point mutation at position 158 that replaces the arginine residue in apoE3 with cysteine, while the ε4 allele encodes the apoE4 isoform with an arginine residue at both positions 112 and 158 [17]. The apoE protein was discovered initially as an arginine-rich protein associated with various plasma lipoproteins, including chylomicron remnants, very low density lipoproteins (VLDL), and a subclass of large high density lipoproteins (HDL). Its presence in these lipoproteins is responsible for their clearance from the plasma circulation via interaction with specific cell surface receptors and heparan sulfate proteoglycans. The apoE in plasma lipoproteins is derived primarily from hepatocytes, but apoE is also synthesized in numerous extrahepatic tissues, including the brain, adipocytes, immune cells of the myeloid lineage, vascular smooth muscle cells, kidney, and the adrenal glands [18]. Thus, apoE has numerous functions in addition to mediating plasma lipoprotein transport in health and diseases. This review article focuses on lipoprotein transport-dependent and -independent mechanisms, by which apoE deficiency or polymorphism contribute to cardiovascular disease, metabolic disease, and neurological disorders. Due to space limitations, we will not review the role of apoE in health and disease of the kidney, including the relationship between minor apoE variants and glomerulopathy. Readers interested in this latter topic are referred to another recent review article on this subject [19].

## 2. Role of ApoE in Cardiovascular Health and Disease

### 2.1. Atherosclerosis

Cardiovascular disease is the leading cause of death worldwide, accounting for approximately 860,000 deaths in the United States and 18.6 million deaths globally in 2019 [20]. Risk factors associated with cardiovascular disease include age, genetic predisposition, gender, smoking, hypertension, stress, and dietary habits [21]. The key underlying cause of cardiovascular disease is atherosclerosis in the coronary arteries. Atherosclerosis is caused by chronic inflammation and lipid accumulation in the arterial wall. The disease progresses from an early lesion, a simple fatty streak, to a more complex and well-established plaque with clinical symptoms, such as hardening of the arteries and obstructed blood flow [22]. Macrophages, smooth muscle cells, lipids and extracellular matrix are found in both early and late stages lesions but in different proportions [22].

Large scale population studies revealed the association between *APOE* polymorphisms with increased risk of coronary and perivascular diseases [2,3,4,5,6,7,8,9]. In particular, carriers of the ε4 allele have a ~1.7-fold increased risk of coronary artery disease [2,23], whereas the relationship between the ε2 allele with cardiovascular disease risk is less clear, with some studies showing the association of ε2 with lower plasma cholesterol levels and reduced risk of coronary heart disease [9,24], while other studies showed an association between the ε2 allele with dyslipidemia and increased risk of ischemic cardiovascular diseases [3,4,5,12,25,26]. Interestingly, the comprehensive Framingham study revealed that after adjustment for age, total- and HDL-cholesterol levels, the odds of developing ischemic heart disease after 12 years is 1.36 for ε2 carriers compared with ε3/3 subjects [27]. Regardless, the ε2 allele is consistently associated with an increased risk of coronary heart disease in diabetes patients compared to ε3/3 diabetics [10,12,15,28,29]. Moreover, heterozygous and homozygous ε2 carriers also have, respectively, a 1.85- and 3.5-fold increased risk for peripheral vascular diseases and carotid artery atherosclerosis in the general population [5], due to mechanisms that are independent of plasma cholesterol levels [26]. The inconsistencies among various studies regarding the importance of ε2 in atherosclerotic disease risk may stem from data collection and analysis without regard to the vascular bed where atherosclerotic lesions occur. Specifically, ε2 is generally associated with lower LDL-cholesterol levels and therefore is not associated with or has a reduced risk of coronary artery disease. However, the ε2 allele is strongly associated with plasma triglyceride levels and elevated postprandial lipemia [30]. These are well-established risk factors for peripheral vascular diseases, including disease of carotid and cerebrovascular arteries, the aorta, and arteries in the lower extremity [31,32]. Therefore, the association between ε2 allele with peripheral vascular disease may be due, at least in part, to its association with plasma triglyceride levels and postprandial hyperlipidemia.

#### 2.1.1. ApoE and Lipid Metabolism

The most well-known function of apoE is its role as the ligand responsible for the clearance of triglyceride- and cholesterol-rich lipoproteins from the plasma circulation, via its high affinity binding to low density lipoprotein (LDL) receptor, the LDL receptor-related protein-1 (LRP1), and heparan sulfate proteoglycans on the surface of hepatocytes (reviewed in [17,33]). This process is initiated by the interaction of basic amino acid residues in the receptor binding domain of apoE, residues 130–150, with negatively charged moieties in the ligand binding domains of these receptors, followed by endocytosis and intracellular degradation of the lipoproteins in the lysosomes.

The importance of apoE-mediated plasma lipoprotein clearance and atherosclerosis was established by the robust hyperlipidemia and atherosclerosis observed in *ApoE^−/−^* mice [34]. While apoE deficiency is rare in humans, the importance of apoE in plasma lipoprotein homeostasis was confirmed in subjects with type III familial hyperlipoproteinemia (dysbetalipoproteinemia), where the elevated plasma triglyceride and cholesterol levels and increased atherosclerosis risk were attributed to the ε2 allele in the *APOE* gene [35]. As noted above, apoE2 differs from apoE3 by an arginine-to-cysteine substitution at residue 158, which is adjacent to the receptor binding domain at residues 130–150. The dysbetalipoproteinemia is generally thought to be due to its impaired LDL receptor binding activity, thereby causing delayed clearance of triglyceride-rich lipoproteins and their remnants. However, most ε2 carriers display only mild hypertriglyceridemia with low or normal cholesterol levels, and the subset of individuals (~15% of ε2/2 subjects) that are predisposed to dysbetalipoproteinemia generally have late onset. These observations imply that additional factors are necessary to complement apoE2 mutation for dysbetalipoproteinemia onset. Secondary risk factors include obesity, diabetes, decreased remnant clearance due to other genetic polymorphisms, increased age and insulin resistance [36]. These observations suggest that the Arg-Cys substitution in residue 158 of apoE2 may not have direct influence on its receptor binding activity, but the conformation of apoE2 in lipoprotein particles may be an important criterion in determining its affinity to interact with cell surface receptors in mediating plasma lipoprotein clearance. Consistent with this hypothesis was the observation that subjects with lysine-to-glutamate mutation within the receptor binding domain, at residue 146, of apoE, displayed a dominant dysbetalipoproteinemia phenotype with an early age onset [37]. Moreover, a case-controlled study showed that the receptor binding activity of apoE2-containing β-VLDL in a homozygous type III familial hyperlipoproteinemia subject was improved to near-normal level with a lipid lowering diet [38].

In contrast to apoE2, apoE4 with arginine residues at both residues 112 and 158 is similar to apoE3 with high affinity binding to the LDL receptor, LRP1, and heparan sulfate proteoglycans. Nevertheless, apoE4 carriers also display higher atherogenic lipoprotein levels in their plasma circulation. Structural characterization of the apoE isoforms revealed that the conformation of apoE4 is different from apoE2 and apoE3. The presence of arginine instead of cysteine in residue 112 of apoE4 results in the formation of a salt bridge with glutamic acid-109, forcing arginine-61 side chain to move away from the 4-helix bundle structure, and allowing it to interact with glutamic acid-255 in the lipid binding region of the apolipoprotein. The interaction between Arg-61 in the N-terminal domain (residues 1–191) and Glu-255 in the C-terminal domain (residues 216–299) alters the conformation of apoE4 to a more compact structure compared to apoE3 [39,40]. As a result of these conformational differences, apoE4 preferentially resides in the triglyceride-rich VLDL and chylomicron remnants, whereas apoE2 and apoE3 preferentially localize to HDL. It is postulated that apoE4 promotes hyperlipidemia because its preference for triglyceride-rich lipoproteins may accelerate their plasma clearance, leading to down-regulation of the LDL receptor and a corresponding increase in plasma LDL and atherosclerosis risk [17].

The importance of N- and C-terminal domain interaction in dictating the preference of apoE for various lipoproteins is further supported by observations made with a minor variant, apoE7, in which both glutamate-244 and glutamate-245 in the C-terminal domain are mutated to lysine residues [41]. These mutations promote N- and C-terminal domain interaction via electrostatic interaction with glutamate-49 and glutamate-50 in the N-terminal domain, thereby resulting in a more compact structure similar to that observed in apoE4 and its preference for binding to VLDL [41,42]. Interestingly, apoE7 also promotes hyperlipidemia and atherosclerosis, but in contrast to apoE4, apoE7 promotes hyperlipidemia and atherosclerosis due to its impairment in binding to the LDL receptor [43].

It is interesting to note that apoE in other species, including mice, is similar to apoE4 with an arginine residue at position 112 [17]. However, these apoE proteins differ from apoE4 and have a threonine residue instead of arginine at position 61 [17]. Arginine-112 does not interact with threonine-61, thus apoE in nonhuman species is structurally similar to human apoE3 instead of apoE4, despite the presence of arginine at position 112. Accordingly, nonhuman apoE are functionally similar to apoE3 and preferentially reside in HDL instead of the larger triglyceride-rich lipoproteins. The concept that the structure of apoE dictates its function is supported by experiments showing that replacing threonine-61 with arginine in mouse apoE recapitulated the structural properties of apoE4 as well as its preferential association with triglyceride-rich lipoproteins [44].

Studies conducted with human *APOE* gene replacement mice, in which the endogenous mouse *ApoE* gene has been replaced by the human *APOE2, APOE3,* or *APOE4* gene, provided additional insights toward understanding how the various major apoE isoforms may influence lipid metabolism and atherosclerosis (reviewed in [34]). However, it is important to note that while results from these studies with human *APOE* gene replacement mice provided some valuable mechanistic information, phenotype observed in these animals did not fully recapitulate the clinical symptoms observed in humans. For example, similar to human ε3/3 subjects, the human *APOE3* mice have normal plasma lipid levels and are relatively insensitive to diet-induced atherosclerosis. However, unlike human ε4 carriers, the *APOE4* mice are normolipidemic and displayed minimal atherosclerotic lesions when fed a Western type diet [45]. Nevertheless, hypercholesterolemia and robust atherosclerosis were observed in *APOE4* mice with transgenic high expression of human LDL receptor [46]. Likewise, while most of the ε2 carriers in humans have normal or low plasma cholesterol levels and have lower risk of coronary atherosclerosis, with only a subset of individuals developing dysbetalipoproteinemia, all human *APOE2* gene replacement mice displayed high plasma cholesterol and triglyceride levels with spontaneous atherosclerosis even on a regular chow diet [47]. The discrepancies of results between mouse and human studies may be due to important differences in lipoprotein metabolism between the two species. These include differences in lipoprotein assembly and modulation, expression of both apolipoprotein B48 and apolipoprotein B100 in mouse liver but only apolipoprotein B100 in human liver, and the lack of plasma cholesteryl ester transfer protein in mice [48]. These differences also raise cautions regarding the exclusive dependence on mouse studies, and highlight the importance of conducting parallel studies in humans before definitive conclusions can be made [48,49].

#### 2.1.2. ApoE and Endothelial Cells

The first line of defense against intravascular lipid deposits and atherosclerotic lesion formation is an intact endothelium. While apoE is not expressed in vascular endothelial cells [50], apoE associated with plasma lipoproteins as well as apoE derived locally by vascular mural cells plays an important role in maintaining endothelial homeostasis throughout the aging process. This was clearly illustrated by studies showing normal endothelial-dependent relaxation in young *ApoE^−/−^* mice, but nitric oxide (NO)-mediated vasodilation was significantly compromised in older *ApoE^−/−^* mice [51]. Importantly, deficiency in NO bioavailability was noted in *ApoE^−/−^* mice prior to the presence of atherosclerotic lesions [52], indicating that the atheroprotective properties of apoE include its maintenance of endothelial function during the aging process. Mechanistically, the extracellular apoE internalized into endothelial cells disrupts the interaction between caveolin-1 and endothelial NO synthase (eNOS) in lipid rafts, thereby preventing caveolin suppression of eNOS activity to increase NO production [53]. The enhancement of eNOS activity also inhibits cell adhesion molecule expression to limit monocyte recruitment and reduces vascular inflammation to prevent vascular occlusion [54]. This process is initiated by apoE interaction with apoE receptor-2 on endothelial cell surface, since apoE activation of eNOS and the reduction of endothelial adhesion of monocytes were not observed in mice with apoE receptor-2 deficiency [54,55]. Interestingly, while apoE receptor-2 has no isoform preference and binds apoE2, apoE3, and apoE4 with similar affinity [56], apoE3 is more efficient than apoE2 and apoE4 in eNOS induction [57]. Moreover, apoE4 antagonizes apoE3 activation of eNOS and ameliorates apoE3 inhibition of monocyte adhesion [54]. The mechanism underlying how apoE4 antagonizes apoE3 effects on endothelial cells remains unknown, but it is unlikely due to competition of receptor occupancy since an order of magnitude lower concentrations of apoE4 was sufficient to inhibit the apoE3 effects [54]. One possibility is that apoE4 inhibits intracellular recycling of apoE receptor-2 to reduce its cell surface level similar to that observed in neural cells [58]. This hypothesis remains to be tested with endothelial cells. Regardless, these data documented that apoE regulates endothelial cell functions in an isoform-dependent manner and established one mechanism by which apoE is protective against vascular occlusive diseases.

#### 2.1.3. ApoE and Macrophages

As noted earlier, atherosclerosis is a chronic disease caused by hyperlipidemia and inflammation of the vasculature due to endothelial cell injury. Monocyte-derived macrophages play an active role in early stages of atherogenesis and are essential constituents of both early phase fatty streak lesions and the more advanced atherosclerotic plaques in human and experimental mouse models [59]. In hyperlipidemia, apoB-containing lipoproteins infiltrate into the subendothelium after endothelial injury, where they are oxidatively modified and endocytosed by infiltrating monocytes/macrophages, leading to the formation of pro-inflammatory lipid-laden foam cells [60]. The inflammatory response activates normally quiescent medial smooth muscle cells and induces their migration from the media to the intima, where they proliferate and synthesize the extracellular matrix. Activation of smooth muscle cells and the accumulation of inflammatory macrophages also trigger infiltration of other innate immune cells, such as neutrophils, natural killer (NK) and NKT cells, as well as an inflammatory adaptive immune response involving T and B lymphocytes [61]. The initial immune response typically dampens inflammation and promotes repair, but under chronic situations when endothelial injury is unabated, sustained maladaptive inflammation will lead to continuous expansion of fibrous materials overlaying the lipid- and foam cell-rich core, resulting in an intermediate lesion. In early lesions, resolution may occur through cholesterol efflux from macrophages to reduce lipid accumulation and inflammation, and phagocytic myeloid cell engulfment of dead cells in a process called efferocytosis [62]. In advanced lesions, impaired cholesterol efflux and inefficient efferocytosis leads to necrotic core expansion to form complex lesions that are prone to rupture [62,63,64,65]. Extracellular apoE associated with lipoproteins in plasma circulation, or derived locally in the vessel wall, as well as apoE expressed endogenously in macrophages participates in each step of this process to limit atherosclerosis progression. ApoE deficiency in *ApoE^−/−^* mice leads to robust atherosclerosis with exacerbated vascular inflammation in addition to the elevated plasma lipid levels caused by impaired lipoprotein clearance [34].

Extracellular apoE inhibits macrophage inflammatory response and limits inflammatory cytokine secretion by inhibiting toll-like receptor (TLR)-3 and TLR-4 mediated c-Jun and c-Jun N-terminal kinase activation. In vitro studies revealed that exogenous apoE binding to heparan sulfate proteoglycans on the macrophage cell surface is responsible for the inhibition of TLR-3 mediated activation, whereas its interaction with LDL receptor related proteins is responsible for the inhibition of TLR4 activation [66]. Additionally, apoE also interacts with the ATP binding cassette transporter ABCA1 to promote intracellular cholesterol efflux [67]. Moreover, exogenously added apoE also increases ABCA1 expression in macrophages via activation of phosphoinositol 3-kinase (PI3K), protein kinase Cξ (PKCξ) and specificity protein-1 (Sp1) [68]. The ability of exogenous apoE to promote ABCA1 expression and activity in macrophages enhances cholesterol efflux and prevents excessive intracellular cholesterol accumulation, thereby reducing cholesterol-induced inflammasome activation [69]. The interaction between apoE and ABCA1 does not show isoform-dependent differences, and apoE2, apoE3, and apoE4 are equally active in promoting ABCA1-mediated cholesterol efflux [70]. However, apoE4 is less efficient in activation of the PI3K-PKCξ-Sp1 pathway to increase ABCA1 expression, thus resulting in the overall lower efficiency of apoE4 compared to apoE3 in promoting macrophage cholesterol efflux [68].

ApoE expressed endogenously in macrophages regulates inflammatory response via multiple mechanisms. Firstly, endogenously expressed apoE promotes cholesterol efflux from macrophages [71,72,73]. In fact, endogenously expressed apoE appears to be more efficient than exogenously added lipid-free apoE in promoting cholesterol efflux from macrophages in vitro [71]. Apparently, endogenously expressed apoE is secreted in conjunction with intracellular cholesterol and phospholipid to form an apoE-lipid complex, which in turn promotes additional cholesterol efflux via interaction with ABCA1 [71]. One study indicated that macrophage-expressed apoE is necessary and systemic apoE is not sufficient to efficiently promote macrophage cholesterol efflux and reverse cholesterol transport [73,74]. However, this study does not rule out the possibility of a two-step mechanism by which apoE expressed endogenously is secreted along with intracellular cholesterol and phospholipids, and the secreted apoE-lipid complex acts in a paracrine manner to promote ABCA1-mediated cholesterol efflux. The separate autocrine and paracrine effects of apoE-mediated macrophage cholesterol efflux is concentration-dependent [72].

ApoE-mediated cholesterol efflux from macrophages is dependent on the isoform produced. The mutation of apoE3 to apoE2 or apoE4 impairs macrophage cholesterol efflux, via mechanisms that are distinct between the two apoE isoforms. While apoE2 expressed in macrophages reduces cholesterol efflux due to impairment of its secretion from macrophages [75,76], expression of apoE4 in macrophages lowers cholesterol efflux due to its inability to stimulate Sp1 signaling and induction of ABCA1 expression [68]. The lower efficiency of macrophage-derived apoE2 and apoE4 to promote cholesterol efflux leads to higher intracellular cholesterol accumulation and foam cell formation. Additionally, the defects in cholesterol efflux also leads to inflammasome activation and the consequential effects of increased inflammatory cytokine production [69]. The increased intracellular cholesterol level also elevates lipid rafts formation, thereby promoting IL3/GM-CSF signaling to enhance hematopoietic stem cell proliferation, and monocytosis [77]. Each of these steps is an important contributor to the atherosclerosis process.

In addition to the role of apoE in regulating macrophage cholesterol homeostasis to limit inflammatory response, a second mechanism underlying the anti-inflammatory properties of macrophage-derived apoE is its enhancement of microRNA-146a synthesis [50], leading to the lowering of tumor necrosis factor receptor associated factor 6 (TRAF6) and interleukin-1 receptor-associated kinase 1 (IRAK1) levels and the resulting suppression of nuclear factor ĸB activation [50]. The underlying mechanism by which apoE enhances miRNA-146a synthesis is indirect, via apoE-induced expression of the transcription factor purine-rich PU-box binding protein 1 (PU.1) [50]. Whether apoE can translocate into the nucleus and directly modulate gene transcription activity as proposed, or acts epigenetically through signal transduction events, needs further clarification. Moreover, whether apoE regulation of PU.1 transcription activity and microRNA-146a synthesis is isoform-dependent remains to be determined.

A third mechanism by which apoE expressed in macrophages is atheroprotective is due to its ability to reduce macrophage-mediated LDL oxidation. Interestingly and in contrast to purified apoE isoforms in which apoE4 was shown to enhance LDL oxidation compared to apoE3 and apoE2 [78], apoE2-expressing macrophages were found to enhance LDL oxidation more avidly compared to apoE3- and apoE4-macrophages [79]. The reduced effectiveness of macrophage-derived apoE2 to suppress oxidation of LDL in the extracellular milieu is likely due to the reduced secretion of this apoE isoform compared to apoE3 and apoE4. The data showing the anti-oxidation properties of apoE4-expressing macrophages are puzzling considering that apoE4 has been shown to promote LDL oxidation in vitro [78]. Other studies also showed increased cell membrane oxidation, more nitric oxide production, and superoxide anion radicals in apoE4-expressing macrophages compared to apoE3-expressing macrophages [80]. Data from our laboratory showing that macrophages with apoE4 expression also displayed elevated endoplasmic reticulum stress and efferocytosis impairment compared to apoE3-expressing macrophages are consistent with the pro-oxidation and pro-atherosclerosis properties of apoE4 expressed in macrophages [81]. The reason for the discrepancy in results regarding the pro- or anti-oxidation role of apoE4-expressing macrophages is unclear.

#### 2.1.4. ApoE and Lymphocyte Activation

ApoE is not expressed in lymphocytes [50]. However, both exogenous apoE and apoE synthesized by myeloid-lineage cells are regulators of lymphocyte activation and adaptive immune response. Earlier studies have shown that apoE and apoE-containing lipoproteins inhibit T lymphocyte proliferation and reduce interleukin-2 activity in vitro [82,83]. The physiological significance of these in vitro observations was noted by studies showing that apoE deficiency dramatically increases susceptibility to endotoxemia and *Klebsiella pneumoniae* infection and disseminated candidiasis in mice [84,85]. Although apoE isoform preference in immunoregulation remains unclear, the overrepresentation of apoE4 in herpes simplex virus-infected individuals [86], and the elevated susceptibility to opportunistic organism infection in HIV-positive patients with the ε4 allele [87], provided evidence to suggest that apoE4 is less effective in suppressing lymphocyte immune response in vivo. Taken together, both the in vitro and in vivo data revealed the ability of apoE to suppress lymphocyte response to inflammation, thereby illustrating another mechanism by which apoE protects against atherosclerosis in an isoform-dependent manner.

**Figure 1:**
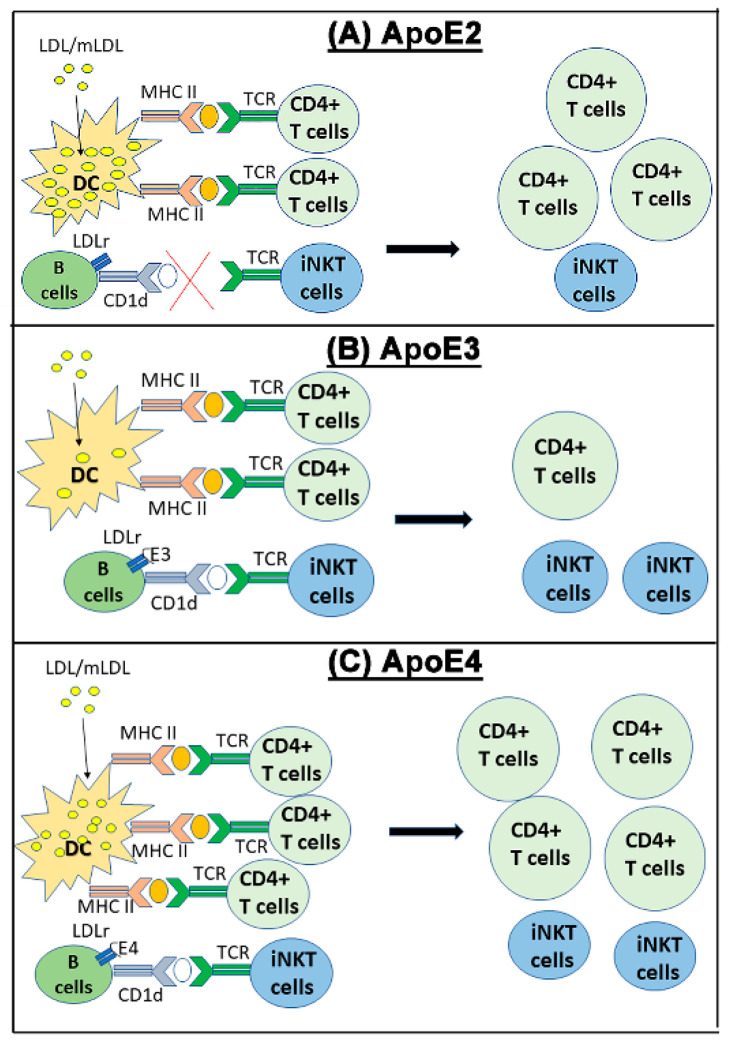
Schematic diagram depicting the influence of various apoE isoforms on antigen presentation. (**A**) ApoE2 enhances intracellular cholesterol accumulation after dendritic cell uptake of LDL/modified LDL to enhance MHC-II expression, thereby promoting antigen presentation to CD4^+^ T lymphocytes for their activation. The defective apoE2 binding to the LDL receptor in B lymphocytes also impairs CD1d-mediated antigen presentation and iNKT cell activation. (**B**) ApoE3 limits intracellular cholesterol levels in dendritic cells, thereby reduces MHC-II expression and T lymphocyte activation. ApoE3 also interacts with the LDL receptor on B lymphocytes to enhance CD1d-mediated antigen presentation to iNKT cells. (**C**) ApoE4 enhances intracellular cholesterol accumulation in dendritic cells to enhance MHC-II expression and antigen presentation for T lymphocyte activation. ApoE4 also interacts with the LDL receptor on B lymphocytes to enhance CD1d-mediated antigen presentation to activate iNKT cells.

ApoE expressed in myeloid-lineage cells, including macrophages and dendritic cells, participates in adaptive immunity indirectly by regulation of antigen presentation. In particular, dendritic cells with apoE deficiency or apoE4 expression showed enhanced MHC-II dependent antigen presentation and activation of pro-inflammatory Th1 CD4^+^ lymphocytes [88]. Hence, apoE4 expressed in myeloid cells may enhance the recruitment and activation of Th1 cells in atherosclerotic lesions to accelerate lesion progression to a more complex phenotype [89]. The mechanism by which endogenous apoE4 expression in myeloid cells increases MHC-II antigen presentation has not been elucidated completely. A potential mechanism may be related to the decreased ability of apoE4 to mobilize intracellular cholesterol compared to apoE3, leading to the enhanced antigen presentation by HLA-DR, and increased polarization of CD4^+^ naïve T cells [88]. In support of this mechanism is the increased lipid rafts content in human dendritic cells expressing apoE4. Interestingly and in contrast to apoE4, apoE2 impairs CD1d-mediated lipid antigen presentation in a paracrine manner due to it defective interaction with the LDL receptor [90,91,92]. Since reduced lipid antigen presentation may limit atherosclerosis advancement toward a more complex stage [91], myeloid-derived apoE2 may accelerate atherosclerosis during the early phase of foam cell formation and deposition, but impedes atherosclerosis advancement to the complex lesion stage similar to that observed in hyperlipidemic *CD1d^−/−^* mice [93]. On the other hand, the defective secretion of apoE2 by macrophages may also lead to impairment in suppression of antigen presentation to enhance atherosclerosis advancement. Although the hypothesis that myeloid cell-derived apoE2 and apoE4 differentially influence early and late stages of atherosclerosis remains to be tested with more direct experiments, we have recently shown that apoE2 and apoE4 expression in myeloid cells influences lymphocyte activation and enhances atherosclerosis via distinct mechanisms [76]. Our data indicate that apoE2 increases macrophage inflammation due to impaired cholesterol efflux and inflammasome activation, whereas apoE4 enhances macrophage inflammation via elevated oxidative stress. A schematic diagram depicting the influence of various apoE isoforms on antigen presentation is shown in Figure 1.

#### 2.1.5. ApoE and Vascular Smooth Muscle Cells

A major cell type present in the vessel wall is the smooth muscle cell. Under normal homeostatic conditions, most vascular smooth muscle cells are quiescent and exhibit a contractile phenotype that provides the biomechanics for vascular contraction. However, exposure to serum factors, such as those observed under conditions of endothelial injury and/or atherosclerosis, leads to their switch to a synthetic phenotype with high migratory and proliferative rates that forms the neointima and atherosclerotic plaque. ApoE is highly expressed in quiescent smooth muscle cells and its expression is dramatically reduced during the proliferative phase [94]. Whether apoE has intracellular functions in smooth muscle cells is unknown, but apoE secreted locally in the vasculature as well as apoE associated with plasma lipoproteins has been shown to play an important role in limiting the expansion of the intravascular smooth muscle cell pool [95]. In vitro studies revealed that exogenous apoE influences smooth muscle cell phenotypes through multiple mechanisms. As noted in an earlier review article [96], apoE inhibits smooth muscle cell proliferation via its interaction with heparan sulfate proteoglycans on the cell surface with subsequent activation of inducible nitric oxide synthase, which leads to cell arrest at the S phase of the cell cycle and the return of cells at the G_1_-S boundary to the quiescent G_0_ state [97]. Additionally, apoE also inhibits smooth muscle cell migration via its interaction with LRP1. This interaction leads to activation of the cAMP/protein kinase A pathway, and inhibition of protein kinase A activity in smooth muscle cells, abolished the effects of apoE on smooth muscle cell migration. Consistent with the affinity of the various apoE isoforms for binding to LRP1 and heparan sulfate proteoglycans [98,99], apoE inhibition of smooth muscle cell migration has no isoform preference, while apoE3 is more effective than apoE2 and apoE4 in suppressing smooth muscle cell growth. The ability of apoE to inhibit smooth muscle cell migration and proliferation may account for its ability to not only suppress hypercholesterolemia-induced atherosclerosis but also to inhibit endothelial denudation-induced neointimal hyperplasia [95,100]. The higher incidence of restenosis after coronary angioplasty observed in ε4 carriers compared to ε3 carriers is consistent with this hypothesis [101,102].

In addition to its role in modulating smooth muscle cell migration and proliferation to suppress vascular occlusive diseases, apoE also plays an important role in maintaining vascular biomechanics under normal conditions. In this regard, apoE deficiency has been shown to increase arterial stiffness in mice due to increased expression of extracellular matrix genes [103]. In vitro studies revealed that extracellular apoE inhibits expression of extracellular matrix genes, most avidly in dedifferentiated smooth muscle cells but only minimally in contractile smooth muscle cells in response to substratum stiffening [96,103]. The resulting effect of these differences is the ability of apoE to suppress the remodeling of the extracellular matrix during atherosclerosis without the adverse effect on normal vascular architecture under normal conditions [103]. Mechanistically, apoE stimulates the expression of cyclooxygenase-2, a stiffness-sensitive inhibitor of extracellular matrix genes [104], and microRNA-145 that targets lysyl oxidase to impede arterial stiffening during aging and limits adverse vascular remodeling during vascular injury and atherosclerosis. Interestingly, apoE suppression of extracellular matrix gene expression shows no isoform preference [103], implying that apoE polymorphism has no influence on vascular remodeling. The lack of association between apoE polymorphism with arterial stiffness in subjects without cardiovascular symptoms is supportive of this interpretation [105].

#### 2.1.6. Summary of ApoE Isoform Influence on Atherosclerosis

**Figure 2:**
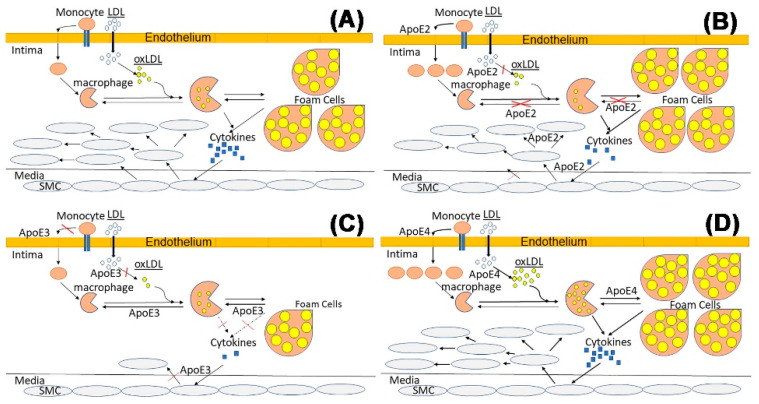
Schematic diagrams on the influence of apoE in atherosclerosis. (**A**) General schematics of the atherosclerosis process. (**B**) Mechanisms underlying the influence of apoE2 in accelerating atherosclerosis. (**C**) Mechanisms underlying the protective roles of apoE3 on atherosclerosis development. (**D**) Mechanisms underlying the influence of apoE4 in accelerating atherosclerosis.

In summary, in addition to its role in modulating plasma lipid homeostasis, apoE also impacts atherosclerosis development and progression via modulating cell functions in a cell type-specific and isoform-dependent manner. In particular, normal apoE function is important for vascular health for protecting against: (i) endothelial dysfunction, (ii) monocyte adhesion to the vessel wall and their infiltration into the subendothelial space, (iii) pro-inflammatory macrophage activation, (iv) macrophage cholesterol accumulation, (v) LDL oxidation, (vi) migration of medial smooth muscle cells into the intima, and (vii) proliferation of smooth muscle cells in the intima. Additionally, a review of the literature reveals that mutations of apoE3 to apoE2 or apoE4 cause impairment at distinct steps in this process to accelerate atherosclerosis (Figure 2).

## 3. Role of ApoE in Metabolic Health and Disease

### 3.1. ApoE and Obesity

Current literature on the relationship between *APOE* polymorphism and obesity risk is controversial. Although a preponderance of data indicates a positive relationship between the ε2 allele and increased risk of obesity across several different populations [15,28,106,107,108], this relationship is not consistently observed [109,110]. While the discrepancy has not been resolved, a potential explanation may be related to apoE2 and plasma lipid levels. The lower affinity of apoE2 for the LDL receptor likely leads to impaired hepatic clearance of triglyceride-rich lipoproteins and the deposition of the excess lipids to adipocytes to promote obesity, similar to that observed in human *APOE2* gene replacement mice [111]. Hence, the relationship between the ε2 allele with obesity may not be evident in ε2 carriers with low plasma lipid levels [112]. Studies with *APOE2* mice did not shed additional light on whether impaired apoE-mediated lipoprotein clearance impacts obesity because these animals displayed hyperlipidemia even under basal dietary conditions [47]. Furthermore, although both *ApoE^−/−^* and human *APOE2* gene replacement mice are more sensitive than wild type mice to diet-induced hyperlipidemia, the *ApoE^−/−^* mice are resistant to diet-induced obesity [113] whereas human *APOE2* gene replacement mice are similar to human ε2 carriers and are more sensitive than wild type mice to develop obesity in response to a high fat diet [111]. Additional studies to determine whether the increased risk of human ε2 carriers to obesity is directly due to impaired lipoprotein clearance are warranted. Nevertheless, one difference between *ApoE^−/−^* mice and *APOE2* mice in response to diet-induced obesity may be due to endogenous expression of apoE in adipocytes. Ex vivo studies with mouse adipose tissues as well as in vitro studies with mature adipocytes showed that endogenously expressed apoE enhances adipocyte triglyceride content [114,115], via a secretion-recapture mechanism where the apoE secreted by adipocytes facilitates adipocyte lipid acquisition from exogenous triglyceride-rich lipoproteins [114,115].

Studies exploring the relationship between the ε4 allele and obesity also yielded inconsistent results. While some studies showed that ε4 is associated with higher body mass index [105,116], other studies showed no difference between ε3 and ε4 or even opposite results with the ε4 allele associated with reduced obesity [106,117,118]. The inconsistencies may be due to a variable presence of confounding factors, such as plasma lipid levels and diabetes in each population. Studies with genetically modified mice showed that human *APOE4* gene expression in adipocytes impairs adipocyte differentiation due to defective activation of peroxisome proliferator-activated receptor-γ [119], leading to a metabolic shift in favor of fatty acid utilization and brown adipose thermogenesis [120]. Whether the lower adiposity observed in selected human ε4 carriers is due to similar metabolic shifts remains to be determined.

### 3.2. ApoE and Diabetes

Similar to the inconsistencies reported in the literature regarding the relationship between *APOE* polymorphism and obesity in humans, whether *APOE* polymorphism has direct influence on insulin resistance and diabetes is also controversial [121]. It is possible that *APOE* polymorphism collaborates with other comorbidity factors, such as obesity, chronic infection, hyperlipidemia, hypertension, and neurodegenerative disorders to exacerbate diabetes progression [14,122,123,124]. In this regard, human *APOE2* gene replacement mice, which are hyperlipidemic with elevated adiposity, also exhibit increased sensitivity to diet-induced hyperglycemia and insulin resistance [111]. The higher hyperglycemic response of the *APOE2* mice compared to the *APOE3* mice is attributed to the combination of increased lipid deposition and elevated macrophage inflammation in adipose tissues to cause insulin resistance [111]. Interestingly, despite the lower adiposity observed in *APOE4* gene replacement mice, the *APOE4* mice are also more insulin resistant with increased sensitivity to diet-induced diabetes compared to *APOE3* mice [125]. Mechanistically, apoE4 promotes diabetes due to impaired adipocyte differentiation and reduced expression of the glucose transporter GLUT4 to cause hyperglycemia [125]. These studies with genetically modified mouse models illustrated distinct mechanisms by which apoE2 and apoE4 may enhance diet-induced insulin resistance and diabetes. It is important to note that these studies examining the influence of apoE polymorphisms on diabetes were conducted with animals fed a high fat diet. Therefore, while high caloric intake and obesity may be a comorbidity factor in the influence of apoE polymorphism on diabetes, whether apoE polymorphism confers diabetes risk in nonobese human subjects is unclear.

## 4. Role of ApoE in Neurological Health and Disease

### 4.1. ApoE and Alzheimer’s Disease

Genome-wide association studies revealed that *APOE* is a genetic risk factor for late onset Alzheimer’s disease [16,126,127,128]. Among the three major *APOE* alleles, ε4 allele increases while ε2 allele decreases Alzheimer’s disease risk compared to the most common ε3 allele [126,129,130]. This is the most common cause of dementia in elderly people, characterized clinically by progressive decline in cognitive and memory functions, followed by behavioral changes and impairment in language function [131,132,133]. Pathologically, Alzheimer’s disease is characterized by neuronal loss, synaptic loss, brain atrophy and inflammation associated with amyloid plaque deposits and formation of neurofibrillary tangles in the brain [131,134,135]. Several excellent review articles with comprehensive discussions on the relationship between *APOE* polymorphisms and Alzheimer’s disease risk have been published recently [136,137]. Herein we provide a brief synopsis highlighting key salient findings on mechanisms by which apoE polymorphisms influence Alzheimer’s disease risk.

#### 4.1.1. ApoE and β-Amyloid

Both human and animal studies showed that β-amyloid (Aβ) levels and amyloid plaque loads in brain are higher in ε4 carriers and lower in ε2 carriers [138,139,140]. Studies with targeted gene replacement mice expressing human apoE4 or apoE3 and corresponding in vitro studies revealed that apoE4 affects several important steps in amyloid cascade. Firstly, apoE4 is less efficient than apoE2 and apoE3 in mediating Aβ clearance through cell surface receptors [141], thus indicating that apoE4 adversely affects blood brain barrier clearance of Aβ peptides from the central nervous system [142,143]. These initial observations were surprising considering that apoE4-containing lipoproteins display similar high affinity as apoE3-containing lipoproteins in binding to the LDL receptor and are more efficient than apoE2-containing lipoproteins. The differences between apoE-mediated Aβ clearance in the brain and lipoprotein clearance in the plasma circulation may be due to receptor preferences for the respective substrates. In particular, Aβ complex with apoE2 and apoE3 is cleared from the brain via both the VLDL receptor and LRP1. In contrast, apoE4 redirects Aβ clearance from the more rapid LRP1-mediated process to the slower VLDL receptor pathway [142]. In addition to the reduced clearance of Aβ, apoE4 is also more effective than apoE3 in stimulating the recycling of the amyloid precursor protein, thereby enhancing Aβ production [144], as well as increasing fibrillation of Aβ_1–40_ to form neuritic plaques [145,146]. Interestingly, while apoE4 expression leads to higher levels of Aβ plaques in the hAPP_FAD_ transgenic mouse model compared to apoE3 expression [147], genetic inactivation of the *ApoE* gene resulted in a dramatic reduction of Aβ deposits and fibrillization in the same mouse model [148]. In this regard, apoE in the brain contributes to the pathogenesis of Aβ accumulation and this process is accelerated in ε4 carriers to increase the risk of amyloid-associated Alzheimer’s disease.

#### 4.1.2. ApoE and Tau

Another hallmark of Alzheimer’s disease is neurofibrillary tangles caused by hyperphosphorylation of the microtubule-associated protein tau. In vitro studies documented that apoE4 expression in neuronal cells abnormally increases tau phosphorylation and promotes neurofibrillary tangles, whereas apoE3 overexpression has no effect [149,150,151,152]. In vivo studies with a tauopathy mouse model confirmed that apoE4 expression increases tau phosphorylation with greater somatodendritic tau redistribution compared to similar mouse models with apoE2 or apoE3 expression [152]. Interestingly, deleting *ApoE* gene expression in the same tauopathy mouse model is protective from tau pathology and brain atrophy, thus indicating that pathological conditions of neurofibrillary tangle formation require apoE, and the apoE4 isoform accelerates the formation of these fibrillary tangles. Mechanistically, apoE4 is thought to escape the secretory pathway and interacts with tau in the cytoplasm to induce its hyperphosphorylation [153,154]. However, it is important to note that there is no difference in tau binding between apoE3 and apoE4 [155]. In contrast, apoE4 has been shown to selectively increase tau phosphorylation via activation of extracellular signal-regulated kinase-1 and -2, as well as isoform-dependent suppression of a protein phosphatase 2A-like tau phosphatase [153,156].

The enhancement of tau phosphorylation by apoE4 is restricted to neurons, and apoE4 expression in astrocytes has no influence on tau phosphorylation [157]. The difference between apoE4 in neurons and astrocytes is that apoE4 in neurons is highly susceptible to proteolysis, resulting in the generation of a truncated C-terminal apoE fragment apoE(Δ272–299) that directly promotes tau phosphorylation [157]. The role of this apoE truncated fragment to enhance tau phosphorylation was documented by studies showing that transgenic expression of apoE(Δ272–299) in mice caused tau hyperphosphorylation, preneurofibrillary tangle formation, learning and memory impairment, and early death [158]. Moreover, this C-terminal truncated apoE fragment was not detected in mice expressing apoE2, where tau phosphorylation and cognitive impairment were also found to be reduced [159]. Whether differences in susceptibility of apoE2 and apoE4 to intraneuronal proteolysis is due to conformational differences (as reviewed above in Section 2.1.1) need to be clarified. In this regard, it is noteworthy that apoE7, with a similar conformation as apoE4 due to N- and C-terminal domain interaction [42], is also associated with memory deficits [42]. In contrast, another minor apoE variant with a V236E mutation in the C-terminal domain is associated with reduced risk of Alzheimer’s disease [160,161]. It is also unclear if the specific effects of apoE4 on tau hyperphosphorylation are due exclusively to the C-terminal truncated fragment, or may also be related to differential interaction of intact apoE4 with cell surface receptor or heparan sulfate proteoglycans, or a consequence of its recognition as a misfolded protein [162].

#### 4.1.3. ApoE and Neuroinflammation

In addition to amyloid plaques and neurofibrillary tangles, increased activation of innate immune response is also observed in the brain of Alzheimer’s disease patients. Cells responsible for immune response in the brain are astrocytes and microglia. ApoE in the central nervous system is synthesized primarily in astrocytes, with low levels of expression in quiescent microglia that can be increased upon activation. In many aspects, the role of apoE and the effects of apoE polymorphism on neuroinflammation are similar to their impact in the periphery on atherosclerotic inflammatory response. For example, apoE4 is less efficient than apoE3 in astrocytic phagocytosis of synapses and microglia phagocytosis of Aβ aggregates [163,164], similar to the impairment of macrophage efferocytosis of dead cells observed in apoE4-expressing macrophages [81]. Likewise, apoE4-expressing microglia also respond more robustly to stimulation with increased secretion of pro-inflammatory cytokines compared to apoE3-expressing microglia [165]. The robust inflammation observed in ApoE4-expressing microglia is driven by increased expression of TREM2 and the TREM2-APOE signaling pathway [166], a major regulator of microglial functions in Alzheimer’s disease [167].

In contrast to the impact of apoE4 on neuroinflammation, the effects of apoE2 on neuroinflammation differ from the apoE2 influence on peripheral inflammation and atherosclerosis. Consistent with the reduced risk of Alzheimer’s disease in ε2 carriers, microglia incubated with apoE2 showed lower TREM2 activation compared to apoE3-incubated microglia [165]. Furthermore, microglia with endogenous apoE2 expression also secrete less pro-inflammatory cytokines in response to activation compared to apoE3- and apoE4-expressing microglia [168]. A potential difference between the effects of apoE2 on microglia and macrophage inflammation may be due to cell type-specific effects on the secretion of apoE2. While apoE2 expressed in macrophages is poorly secreted [75], therefore leading to impaired cholesterol efflux and inflammasome activation to accelerate atherosclerosis [76], apoE2 expressed in microglia is more efficiently secreted compared to apoE3 and apoE4 during inflammation [169]. The increased levels of apoE secreted by apoE2-expressing microglia may suppress inflammation in a paracrine manner to protect against Alzheimer’s disease.

#### 4.1.4. ApoE and Neurotoxicity

Another hallmark of Alzheimer’s disease is neuronal cell death that causes cognitive impairment. The expression of apoE in astrocytes is protective against excitotoxicity, regardless of the apoE isoform expressed [170]. In contrast, apoE4 expression in neurons is not protective but causes neuronal loss [170]. Interestingly, the deletion of apoE4 in the neurons of APOE4 gene replacement mice is protective against neuronal cell death [171], suggesting that the apoE4 effect on neuronal cell death is not a result of apoE dysfunction but represents a gain-of-function specific for the apoE4 isoform. As noted previously (Section 4.1.2), apoE4 expressed in neurons is highly susceptible to neuron-specific intracellular proteolysis. The accumulation of the C-terminal apoE4 fragment not only increases tau phosphorylation but also promotes neuronal cell death [157]. The cytotoxic properties of the apoE4 fragment are due to its high affinity binding to and inactivation of complex III and complex IV of the mitochondrial respiratory chain, thereby causing energy deficits and cell death [172,173]. The cytotoxic apoE fragment is not observed in apoE3 or apoE2 expressing neurons. It is unclear whether neuronal proteolysis is specific for the apoE4 isoform or if the proteolytic fragments of apoE3 and apoE2 are rapidly removed without intracellular accumulation.

### 4.2. ApoE and Parkinson’s Disease

In addition to Alzheimer’ disease, apoE also plays a role in other neurodegenerative diseases, such as Parkinson’s disease. The major clinical features of this disease include motor symptoms, such as static tremor, myotonia, postural balance disturbance, and non-motor symptoms, such as sleep disorders, disturbances in olfactory sensation, and autonomic dysfunction [174]. Neuropathological features include the loss of dopaminergic neurons in the substantia nigra and the existence of Lewy bodies (LB) in surviving neurons [175]. In contrast to Alzheimer’s disease with accumulation of Aβ aggregates and tauopathy, fibrils in Parkinson’s disease are associated with the accumulation of α-synuclein inclusions. Nevertheless, the ε4 allele is also associated with increased risk of Parkinson’s disease, suggesting that apoE4-associated proteinopathies may be the common denominator of both of these neurodegenerative disorders. However, while the ε2 allele is associated with lower risk of Alzheimer’s disease, the ε2 allele is not protective against Parkinson’s disease [174]. The mechanistic relationship between apoE4 and α-synuclein fibrils in Parkinson’s disease is less well studied. It is noteworthy, however, that inactivation of the apoE gene also alleviates α-synuclein neurodegeneration in mice [176]. Thus, the apoE4 effects on Parkinson’s disease are also due to its gain-of-function property instead of a dysfunctional apoE protein. One possibility is that apoE4 may enhance α-synuclein activation of extracellular signaling pathways to accelerate neurodegeneration [176]. Another possibility is that apoE4 reduces extracellular α-synuclein uptake and clearance by oligodendrocytes to enhance its aggregation [177]. Additionally, apoE4 expressed in dopaminergic neurons may also be fragmented and exacerbates mitochondrial dysfunction caused by α-synuclein mutation to accelerate brain pathology. These potential mechanisms are not mutually exclusive and may act in concert to increase the risk of Parkinson’s disease in ε4 carriers. Additional mechanistic studies are worthwhile to test these possibilities.

### 4.3. Summary of ApoE Isoform Influence on Neurological Health and Diseases

In summary, the ε4 allele is associated with higher risk of late-onset Alzheimer’s disease and other neurological disorders, such as Parkinson’s disease. In contrast, the ε2 allele is associated with lower risk of Alzheimer’s disease but has no influence on Parkinson’s disease. The current literature indicates that apoE4 increases the risk of neurological disorders by enhancing amyloid accumulation, promoting tau hyperphosphorylation, and exacerbating neuroinflammation in the brain. Genetically modified mouse models revealed that a number of these properties can be ameliorated by ApoE gene inactivation. These observations led to the suggestion that apoE4 reduction may be a promising therapeutic strategy for Alzheimer’s disease management [178]. However, specific apoE reduction in the brain without affecting apoE level and its beneficial properties peripherally would be an enormous challenge. Considering the emerging literature indicating that the detrimental properties of apoE4 may be related to N- and C-terminal domain interaction, and/or the generation of a C-terminal truncated apoE fragment, the use of small molecules that disrupt apoE4 domain interaction or inhibit the neuron-specific apoE proteolysis may be viable therapeutic options for Alzheimer’s disease treatment. Several apoE4 structure correctors have been identified and these molecules have shown promising results in preclinical models [179].

## 5. Conclusions and Perspective

This review highlighted the role of apoE in cardiovascular, metabolic, and neurological health and diseases. In particular, we reviewed literature that showed how apoE deletion and/or polymorphism contribute to disease pathogenesis (Summarized in Table 1). The key features underlying the role of apoE in health and diseases include: (i) its function as a ligand transport protein, (ii) its function as a signaling molecule via binding to cell surface receptors and proteoglycans, (iii) its intracellular role in modulation of cell homeostasis, and (iv) its role in regulation of inflammatory response. Mechanistic insights gained from these studies may be harnessed to optimize treatment strategies for subjects with a high risk of cardiometabolic and neurological diseases due to APOE gene polymorphism. Several approaches, such as the use of apoE mimetic peptides [180], apoE4 structural correctors [179], modulators of apoE trafficking pathways [181], apoE antisense oligonucleotides [182] and APOE gene therapy [183] have been proposed and tested in preclinical models with various efficacies. The challenge of these approaches is the specific targeting to appropriate tissues and cell types, where their effectiveness can be optimized without any adverse effects.

Finally, it needs to be emphasized that despite the preponderance of evidence showing the importance of apoE functions in health maintenance, and how its deletion in mice or polymorphisms in human accelerates disease pathogenesis, apoE is not required for development and apoE-deficient mice survive and develop to maturity without major health issues. The deleterious effects of apoE dysfunction are revealed at later stages after exposure to environmental factors and with different lifestyle choices [184]. Taken together, these observations infer the presence of alternative pathways that serve similar functions as apoE and suggest that apoE is a genetic modifier that acclerates chronic metabolic diseases.

## Figures and Tables

**Table 1:** Influence of apoE inactivation, apoE2, and apoE4 on cardiometabolic and neurological diseases in mice and humans.

Conditions	*ApoE*^−/−^ Mice	*APOE2* Mice	*APOE2* Humans	*APOE4* Mice	*APOE4* Humans
**Cardiovascular**					
Atherosclerosis	↑↑	↑	↑↓ depends on plasma lipids	↑	↑
Plasma lipids	↑↑	↑	↑↓ with confounding factors	↑	↑
Inflammation	↑↑	↑	↑↓ depends on plasma lipids	↑	↑
**Metabolic diseases**					
Obesity	↓	↑	↑↓ with confounding factors	↓	↑↓ controversial
Diabetes	↑	↑	↑	↑	↑
**Neurodegenerative diseases**					
Cognition	↓	↑	↑	↓	↓
Aβ accumulation	↓	↓	↓	↑	↑
Tau phosphorylation	↓	↓	↓	↑	↑
Neuroinflammation	↑	↓	↓	↑	↑
Neuronal toxicity	-	-	-	↑	↑
Parkinson’s disease	↓	-	-	↑	↑

## Data Availability

Not applicable.

## Abbreviations

The following abbreviations are used in this manuscript:

ApoE	Apolipoprotein E
VLDL	Very low density lipoproteins
HDL	High density lipoproteins
LDL	Low density lipoproteins
LRP1	LDL receptor related protein-1
NO	Nitric oxide
eNOS	Endothelial nitric oxide synthase
NK	Natural Killer
NKT	Natural killer T cells
TLR	Toll-like receptor
ABCA1	ATP binding cassette subfamily member 1
PI3K	Phosphatidylinositol 3-kinase
PKCξ	Protein kinase C-ξ
Sp1	Specificity protein-1
IL3	Interleukin-3
GM-CSF	Granulocyte-macrophage colony stimulating factor
TRAF6	TNF receptor associated factor 6
IRAK1	Interleukin 1 receptor associated kinase 1
MHC-II	Major histocompatibility complex II
CD4	Cluster of differentiation 4
CD1b	Cluster of differentiation 1b
cAMP	Cyclic adenosine monophosphate
GLUT4	Glucose transporter type 4
Aβ	Beta-amyloid
TREM2	Triggering receptor expressed on myeloid cells-2
hAPPFAD	human amyloid-beta precursor protein familial Alzheimer’s disease
Th1	T helper cell type 1
HLA-DR	Human leukocyte antigen—DR isotype
